# Targeting radiotherapy-induced inflammation in cancer metastasis: insights into immune modulation, therapeutic opportunities and radiogenomics

**DOI:** 10.3389/fonc.2025.1682522

**Published:** 2025-10-23

**Authors:** Ee Qian Lee, Chin-King Looi, Lu Ping Tan, Yik Ling Chew, Wei-Meng Lim, Lian-Chee Foong, Chee-Onn Leong, Kok Whye Cheong, Chun-Wai Mai

**Affiliations:** ^1^ Faculty of Pharmaceutical Sciences, UCSI University, Cheras, Kuala Lumpur, Malaysia; ^2^ Centre for Cancer and Stem Cell Research, Institute for Research, Development and Innovation (IRDI), IMU University, Bukit Jalil, Kuala Lumpur, Malaysia; ^3^ Molecular Pathology Unit, Cancer Research Centre, Institute for Medical Research, National Institutes of Health, Ministry of Health Malaysia, Shah Alam, Selangor, Malaysia; ^4^ School of Pharmacy, Monash University Malaysia, Jalan Lagoon Selatan, Bandar Sunway, Subang Jaya, Selangor, Malaysia; ^5^ State Key Laboratory of Systems Medicine for Cancer, Renji-Med X Clinical Stem Cell Research Center, Ren Ji Hospital, School of Medicine, Shanghai Jiao Tong University, Pudong New District, Shanghai, China; ^6^ AGTC Genomics Sdn. Bhd, Kuala Lumpur, Malaysia

**Keywords:** radiation, inflammation, immune, therapeutics, radiogenomics

## Abstract

Radiotherapy (RT) is the first-line treatment for more than 50% of newly diagnosed cancer patients and remains a cornerstone of cancer therapy, particularly for tumors that are inoperable, recurrent, or incompletely resected. Despite advancements in RT techniques, locoregional recurrence and distant metastasis remain critical clinical challenges, contributing significantly to cancer-related inflammation and mortality. Emerging evidence suggests that RT may inadvertently promote metastasis through inflammation-related immune modulations, such as the dysregulation of signaling cascades, such as focal adhesion kinase (FAK), phosphoinositide 3-kinases (PI3K)/protein kinase B (AKT), p38 mitogen-activated protein kinase (p38 MAPK), and nuclear factor-kappa B (NF-κB) signaling cascades. Targeting these pro-metastatic pathways using specific inflammatory inhibitors and clinically available repurposed drugs has shown promise in numerous preclinical models, offering a rational approach to mitigate radiation-induced inflammation in metastatic progression. With the rapid advancements of high-throughput sequencing and medical imaging technologies, radiogenomics, which incorporates medical imaging and genomic data, offers great promise for cancer diagnosis, tumor classification, treatment selection, and disease monitoring through the identification of predictive and prognostic biomarkers. This review critically unravels the immune modulation underlying radiation-induced inflammation in cancer metastasis and highlights the need for comprehensive studies combining radiogenomics with RT and targeted therapies. Such approaches hold potential to improve therapeutic outcomes and reduce metastatic burden, paving the way for more effective and personalized cancer treatments.

## 1 Introduction

Radiotherapy (RT) has remained a crucial treatment modality for patients with incompletely resected tumors, recurrent disease, or tumors located in inoperable areas since its development in the late 19th century, following Wilhelm Conrad Roentgen’s discovery of X-rays in 1895 (1–4). Over the years, RT has advanced significantly through continuous technological innovation aimed at improving treatment efficacy while reducing the severe acute and late toxicities associated with conventional two-dimensional RT (2D-CRT) (5). Modern RT techniques, such as intensity-modulated radiotherapy (IMRT), stereotactic body radiotherapy (SBRT), and image-guided radiation therapy (IGRT), enable the precise delivery of high-dose radiation to tumor sites, typically ranging from 60-100 Gy, depending on the modality and fractionation schedule, thereby enhancing tumor control while minimizing damage to surrounding healthy tissues (6–8). To further enhance its therapeutic efficacy, recent studies have explored combining RT with other therapies, including chemotherapy, targeted therapy, immunotherapy, and radionuclide therapy (9, 10). Today, more than 50% of all cancer patients receive RT at some point during their illness, underscoring its critical role in cancer management (11).

While RT is well known for activating cytotoxic signaling pathways and mediating immunostimulatory effects to promote cancer cell death, cancer recurrence, and distant metastasis continue to pose a major challenge in the medical field (2, 12). Studies indicate that nearly 40% of head and neck cancer patients treated with RT experience locoregional recurrence, which is a leading cause of cancer-related mortality (2). Raab et al. (2024) analyzed data from the National Cancer Database (2004–2018) and reported a 90-day mortality rate of 3.5% among head and neck cancer patients undergoing RT (13). One key factor driving recurrence is the intrinsic biological properties of the tumor cells, including inherent sensitivity to RT and the development of acquired resistance (14). A previous study evaluating recurrence and metastasis patterns in nasopharyngeal carcinoma (NPC) patients treated with IMRT found that most patients who experienced local recurrence had received high-dose RT (70–74 Gy), compared to those who received low doses (54–66 Gy) (14). These findings suggest that increasing the radiation dose does not effectively prevent recurrence; instead, it may potentially lead to the development of tumor resistance, further complicating therapeutic outcomes (14). In addition, genomic instability, epigenetic modifications, and variations in the tumor microenvironment (TME) can collectively contribute to tumor heterogeneity, which plays a significant role in mediating RT resistance, enabling tumor cells to adapt and survive (15).

Accumulating evidence suggests that RT may also inadvertently promote the recruitment of tumor cells into the circulation by damaging tumor vasculature, thereby facilitating distant metastasis (16). Metastasis typically begins when tumor cells acquire an invasive and migratory phenotype via the activation of epithelial–mesenchymal transition (EMT), enabling them to detach from their primary site and invade surrounding tissues (4). These cells then enter the bloodstream and travel to distant organs (4). Once in circulation, circulating tumor cells can either metastasize to other sites or reinfiltrate their tumor of origin (4). Although von Essen has outlined four possible mechanisms influencing the rate of metastasis following RT since 1991: radiation-induced biological and behavioral changes in surviving tumor cells; effects on distant normal tissue that may support metastasis; radiation-induced release of tumor cells into the circulation; and the prolonged release of metastatic cells into the circulation due to delayed tumor progression, the molecular mechanisms underlying RT-induced metastasis remain poorly understood (17).

Therefore, gaining a deeper understanding of the mechanisms underlying treatment resistance and RT-induced metastasis is essential for overcoming these significant obstacles in RT. By investigating the molecular and cellular pathways involved, it holds promise for uncovering new therapeutic targets that could enhance the effectiveness of RT and ultimately improve patient outcomes (18). However, translating these experimental findings into clinical applications remains a major challenge, particularly in identifying biomarkers that can predict therapeutic responses and developing targeted interventions capable of preventing or reversing radiation-induced metastasis 19.

Although biopsy is the standard research approach, it provides only a localized representation of the tumor, failing to account for intratumoral heterogeneity and the broader TME, both of which play critical roles in disease progression (19). Furthermore, the identification of clinically relevant biomarkers is complicated by genetic variability, necessitating large-scale studies to ensure their predictive reliability (19). These limitations underscore the urgent need for accurate and cost-effective predictive models to enhance cancer prognosis and optimize treatment strategies.

This review critically discusses the inflammation in metastasis pathways induced by RT, focusing on how these immune-related inflammations contribute to cancer progression. It also explores the use of repurposed and investigational drugs in combination therapies designed to counteract radiation-induced metastasis. In addition, emerging trends in radiomics and radiogenomics are discussed, with a focus on their potential to identify predictive biomarkers for cancer diagnosis and treatment optimization. The review aims to support the development of personalized treatments for cancer patients.

## 2 Radiation-induced inflammation in metastasis

Inevitably, radiation could induce inflammation, leading to immune modulation via four key inflammatory signaling pathways, namely (i) focal adhesion mechanism, (ii) phosphoinositide 3-kinase (PI3K)/protein kinase B (AKT) activation, (iii) p38 mitogen-activated protein kinase (p38 MAPK) activation, and (iv) nuclear factor-kappa B (NF-κB) activation. Radiation-induced focal adhesion activation has been observed in prostate, breast, and colon cancers, while radiation-induced PI3K/AKT activation has been reported in NPC (20–23). Radiation-induced p38 MAPK activation has been reported in hepatocellular carcinoma (HCC), glioma, lung, and cervical cancers, while the radiation-induced NF-κB inflammatory activation has been demonstrated in esophageal, lung, and non-small-cell lung cancers (NSCLC) (24–28). A recent study further suggests that RT can alter the TME, promoting resistance to therapy primarily through the upregulation of inflammatory cytokines such as transforming growth factor-beta (TGF-β) and activation of the AKT, NF-κB, and signal transducer and activator of transcription (STAT) signaling pathways (29). These changes enhance cell proliferation, oxidative stress, M2 macrophage polarization, and angiogenesis, collectively contributing to tumor progression. 30.

### 2.1 Radiation-induced inflammatory focal adhesion mechanism

The extracellular matrix (ECM) is a non-cellular, crosslinked network of macromolecules, including collagens, fibronectins, proteoglycans, and glycoproteins, that provide structural and biochemical support to surrounding cells (30). However, tumor cells can hijack ECM remodeling by altering its composition and organization to facilitate invasion and migration (31). This process is tightly regulated by focal adhesion proteins (31). Focal adhesions are dynamic multi-protein complexes that function as mechanosensors, linking the intracellular actin cytoskeleton to the ECM via integrins (32). Integrins are key cell adhesion proteins that mediate both outside-in and inside-out signaling, and an increase in ECM stiffness activates integrin-mediated mechanosignaling, promoting cell proliferation and local tumor cell invasion (31, 33). Furthermore, integrin binding to ECM exerts mechanical forces that alter ECM conformation, influence matrix degradation and deposition, and promote cancer cell migration even in the absence of proteolytic activity (31). Thus, the natural biomechanisms of ECM remodeling and integrin-mediated focal adhesions provide the basis for tumor invasion, wherein radiation can further modulate these processes.

Over the years, numerous studies have reported that RT can activate focal adhesion mechanisms, leading to the upregulation of integrins and Ras homolog family member A (RhoA), both of which are key regulators of cell adhesion and migration (20–22, 34). For instance, You and co-workers (2021) demonstrated that cell motility mechanisms, particularly focal adhesion, are key pathways in promoting tumor invasion and radioresistance in head and neck cancer (35). Notably, integrin alpha-6 (ITGA6), a heterodimeric component of the integrin receptor protein, was significantly upregulated in tumor cells (35). Subsequent shRNA-mediated knockdown of *ITGA6* expression led to reduced migration and invasion, as well as increased reactive oxygen species (ROS) production (35). These findings suggest that tumor cells may upregulate *ITGA6* to promote cell migration and invasion through ECM integrity modulation and facilitate radioresistance by reducing intracellular ROS levels (35). Therefore, the inhibition of *ITGA6* warrants further studies to determine whether its inhibition could synergize with RT to suppress the migration and invasion of radioresistant cells, as well as re-sensitize tumors to irradiation.

**Figure 1:**
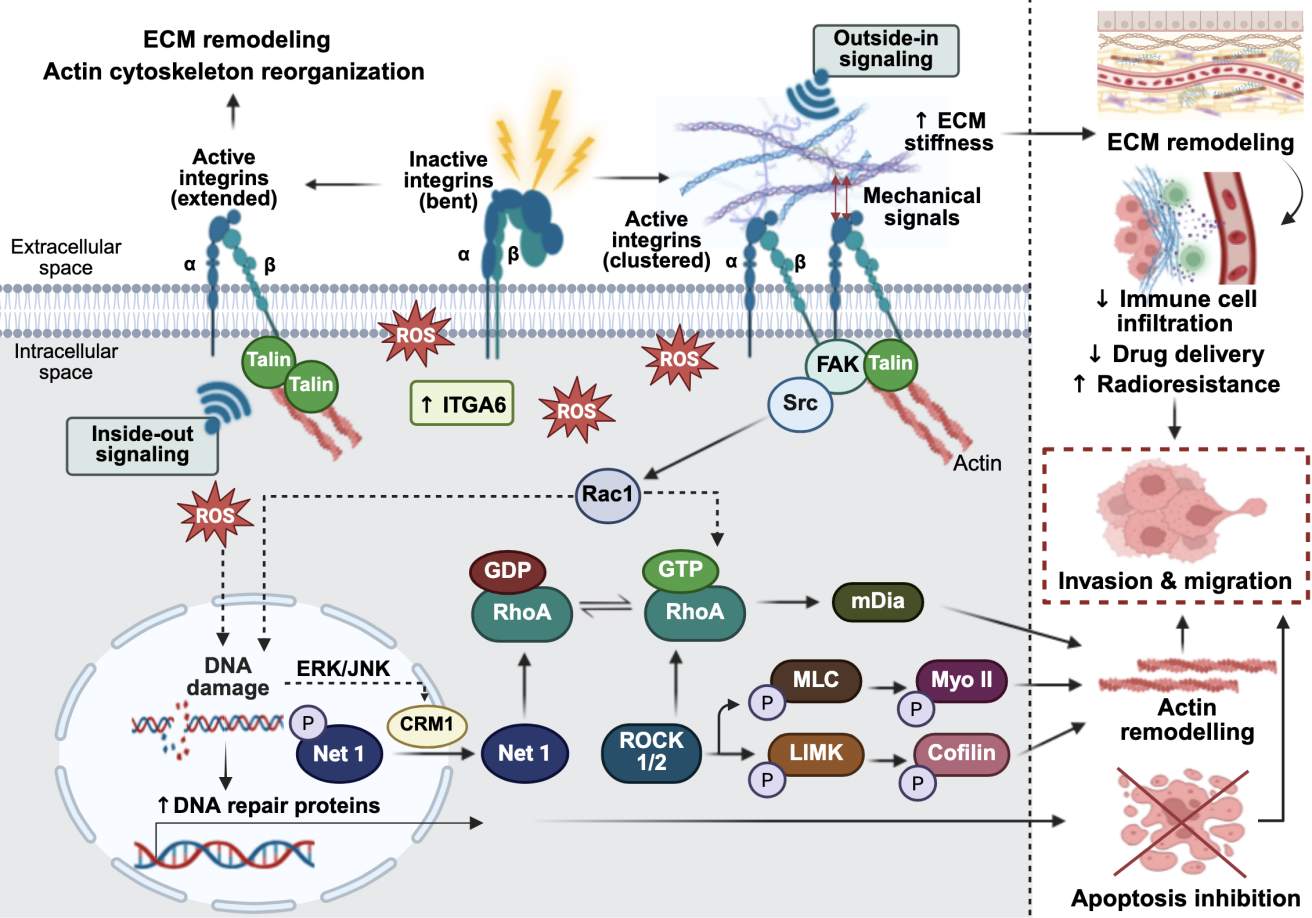
Radiation-induced extracellular matrix (ECM) remodeling, cell migration through focal adhesion signaling and cytoskeletal remodeling leading to cancer immune modulation. In the absence of stimulation, integrins adopt an inactive, closed, bent conformation. Upon exposure to ionizing radiation, reactive oxygen species (ROS) are generated, stimulating inside-out integrin signaling through talin, which reorganizes the actin cytoskeleton and contributes to ECM remodeling. Radiation also upregulates integrin alpha-6 (ITGA6), altering ECM integrity to promote cell invasion. Concurrently, the outside-in binding of ECM ligands to cell surface integrins triggers mechanosignaling that activates focal adhesion kinase (FAK). FAK then associates with proto-oncogene tyrosine-protein kinase Src, leading to activation of the RhoA/ROCK signaling axis. In tumor cells, radiation-induced ROS can cause DNA damage while simultaneously upregulating DNA repair proteins, thereby inhibiting apoptotic cell death and promoting cell survival. The ROS-induced DNA damage response also leads to the dephosphorylation of neuroepithelial cell transforming gene 1 (Net1), a RhoA-specific guanine nucleotide exchange factor (GEF), resulting in its nuclear export via extracellular signal-regulated kinase/c-Jun N-terminal kinase (ERK/JNK)-mediated chromosome region maintenance 1 (CRM1) signaling. Once in the cytoplasm, NET1 activates RhoA, which in turn activates Rho-associated coiled-coil containing protein kinase ½ (ROCK1/2) and the mammalian diaphanous-related formin (mDia). This cascade promotes myosin II contractility and actin filament stabilization, leading to cytoskeletal remodeling. Collectively, these radiation-induced signaling events drive tumor cell migration and invasion. The figure was created using BioRender (https://BioRender.com).

Furthermore, RhoA, the small guanosine triphosphate-binding protein (GTPase), is closely associated with the regulation of cytoskeletal reorganization and vascular permeability after radiation exposure (36). Under normal physiological conditions, RhoA functions as a molecular switch, cycling between an inactive guanosine diphosphate (GDP)-bound state and an active guanosine triphosphate (GTP)-bound form (37). Activation of RhoA is tightly regulated by guanine nucleotide exchange factors (GEFs), which promote the release of GDP from inactive RhoA, allowing GTP to bind and activate it (37). Conversely, Rho GTPase-activating proteins (RhoGAPs) promote the hydrolysis of GTP to GDP, returning RhoA to its inactive state (37). Upon irradiation, DNA double-strand breaks (DSBs) are induced, triggering the DNA damage response (DDR) (38). This signaling cascade, particularly through the epidermal growth factor (EGF) or c-Jun N-terminal kinase (JNK) pathways, promotes the chromosome region maintenance 1 (CRM1)-dependent nuclear export and cytoplasmic translocation of neuroepithelial cell transforming gene 1 (*NET1*) (39). *NET1*, a RhoA-specific GEF, catalyzes the exchange of GDP for GTP, converting RhoA into its active form (39). Activated RhoA subsequently interacts with Rho-associated coiled-coil containing kinases (ROCK; comprising ROCK1 and ROCK2) by binding to their Rho-binding domain (RBD) (40). This binding induces a conformational change that relieves autoinhibition, leading to ROCK activation (40). Once activated, ROCK phosphorylates the myosin light chain (MLC), thereby enhancing myosin II (Myo II) contractility (40). Additionally, ROCK activates LIM domain kinase (LIMK), which phosphorylates and inactivates cofilin, leading to the stabilization of actin filament (37). In parallel, RhoA also activates formin proteins such as the mammalian homolog of diaphanous (mDia), facilitating linear actin polymerization and stabilization (37). The coordinated activities of ROCK and mDia promote the formation of filamentous-actin stress fiber and the assembly of focal adhesion proteins, potentially enhancing the invasiveness of tumor cells following radiation exposure (37). Taken together, these findings position radiation-induced RhoA activation as a critical molecular link between DDR signaling and cytoskeletal remodeling (38). **Figure 1** provides an overview of how irradiation-induced integrin activation and RhoA-mediated cytoskeletal remodeling promote tumor cell invasion and migration.

### 2.2 Radiation-induced inflammatory PI3K/AKT via PNUTS

Protein phosphatase 1 nuclear-targeting subunit (PNUTS), originally identified as a nuclear protein, has been found to modulate the PI3K/AKT pathway, a signaling cascade frequently implicated in cancer progression and cell survival (23, 41). Under normal cellular conditions, PNUTS forms a stable complex with protein phosphatase 1 (PP1), thereby regulating its catalytic activity (42). As a key modulator of PP1, PNUTS participates in various PP1-mediated cellular processes, including transcriptional regulation, cell cycle, apoptosis, and DNA repair (42). Upon exposure to cellular stress, such as hypoxia or radiation, PNUTS dissociates from PP1, allowing PP1 to dephosphorylate the retinoblastoma (Rb) protein, an essential regulator of cell proliferation and apoptosis (43). This dephosphorylation promotes apoptosis, inhibits irradiation-induced EMT, reduces cell viability, and ultimately contributes to tumor suppression (43).

In contrast, overexpression of PNUTS following high-dose irradiation may paradoxically reverse its tumor-suppressive function and potentially contribute to tumor progression, as demonstrated *in vitro* studies conducted by Yu and colleagues (2019) using a 4 Gy dose (23). The study demonstrated that radiation significantly upregulated PNUTS expression in the CNE-2, NPC cell line, and this increase was significantly associated with enhanced migratory and invasive capacities, suggesting that PNUTS overexpression may promote more aggressive tumor behavior following irradiation (23). Further mechanistic studies revealed that PNUTS induces EMT to accelerate CNE-2 cell progression through the PI3K/AKT signaling pathway (23). However, how PNUTS activates the PI3K/AKT pathway was not elucidated in the study.

**Figure 2:**
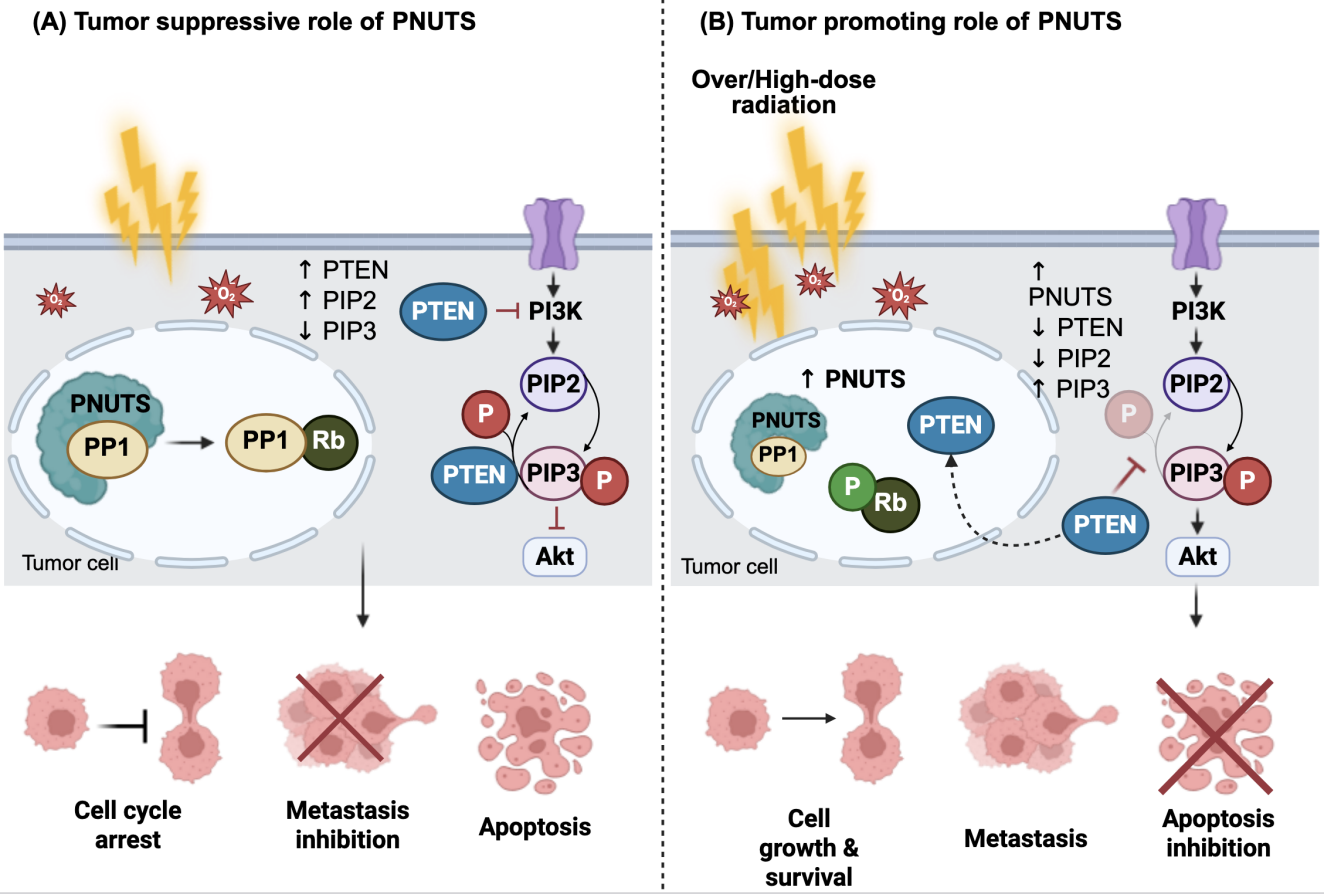
The dual roles of PNUTS in cancers. **(A)** Under basal conditions, protein phosphatase 1 nuclear-targeting subunit (PNUTS) is predominantly localized in the nucleus, where it associates with protein phosphatase 1 (PP1) to regulate its activity. In response to irradiation-induced reactive oxygen species (ROS), PNUTS dissociates from PP1, allowing PP1 to dephosphorylate the retinoblastoma tumor suppressor protein (Rb). This dephosphorylation induces cell cycle arrest and promotes apoptosis, thereby suppressing metastasis. **(B)** In contrast, high-dose irradiation leads to PNUTS upregulation and increased binding to PP1, maintaining Rb in a phosphorylated (inactive) state. Additionally, PNUTS binds directly to phosphatase and tensin homolog (PTEN), sequestering it in the nucleus. This prevents PTEN from dephosphorylating phosphatidylinositol 3,4,5-trisphosphate (PIP3) to phosphatidylinositol 4,5-bisphosphate (PIP2), resulting in cytoplasmic accumulation of PIP3 and constitutive activation of the phosphoinositide 3-kinase (PI3K)/protein kinase B (Akt) signaling pathway. This, in turn, promotes tumor cell growth and metastasis. The figure was created using BioRender (https://BioRender.com).

Previous studies reported that PNUTS binds directly to the lipid-binding C2 domain of phosphatase and tensin homolog (PTEN) via its N-terminal TF2S domain, resulting in the sequestration of PTEN in the nucleus and thereby preventing it from inhibiting PI3K/AKT pathway activation (44). In general, PTEN functions by dephosphorylating phosphatidylinositol 3,4,5-trisphosphate (PIP3), a critical docking site for 3-phosphoinositide-dependent protein kinase-1 (PDK1) and AKT, converting it back to phosphatidylinositol 4,5-bisphosphate (PIP2) (45, 46). This results in reduced PIP3 levels, preventing AKT activation, and thereby inhibiting PI3K/AKT downstream signaling (45, 46). Notably, fractionated ionizing radiation (IR) has been shown to suppress PTEN expression and activate PDK1 and AKT signaling, leading to elevated levels of EMT-related proteins in human esophageal cancer cells (47). Thus, it is suggested that the PNUTS overexpression in response to radiation exposure stimulates PI3K/AKT pathway activation to induce EMT, primarily through the regulation of PTEN activity (23). **Figure 2** illustrates the dual functions of PNUTS in regulating cancer development and progression, highlighting its roles in both tumor suppression and promotion.

### 2.3 Radiation-induced inflammatory p38 MAPK pathway

MAPKs are a family of serine/threonine protein kinases that mediate signal transduction from the cell surface to the nucleus in response to various extracellular stimuli. The conventional MAPK superfamily comprises extracellular signal-regulated kinases (ERK), JNK, and p38 MAPK (48), playing a pivotal role in regulating a wide range of cellular processes, including immune activation, proliferation, differentiation, apoptosis, motility, and stress adaptation (48). Increasing evidence implicates these canonical MAPK pathways as central regulators of cancer cell migration and metastasis. For example, external stimuli such as chemotherapeutic agents and IR can activate the rat sarcoma virus oncogene (Ras)/rapidly accelerated fibrosarcoma (Raf)/mitogen-activated protein kinase (MEK)/ERK cascade, thereby enhancing the migratory capacity of HCC cells (49). Similarly, activation of the JNK/cellular jun proto-oncogene (c-JUN)/matrix metalloproteinase 9 (MMP9) signaling axis facilitates ECM degradation, promoting metastasis in HCC (50). In radioresistant cervical cancer, Kirsten rat sarcoma viral oncogene homolog (KRAS)–driven cell migration has been linked to the activation of the cellular-Raf (c-Raf)/p38 MAPK pathway (51). Collectively, these findings underscore the roles of ERK, JNK, and p38 as key mediators of radiation-enhanced tumor cell migration and invasion.

Among these, p38 MAPK has emerged as a particularly important mediator of radiation-induced tumor aggressiveness. In HCC, residual cancer cells following radiation exhibit increased p38 MAPK activity, which promotes EMT, migration, and invasion. Notably, this effect appears to be driven by hydrogen sulfide (H_2_S), a gaseous neurotransmitter (24). H_2_S is endogenously produced via the transsulfuration pathway, primarily by the catalytic activity of enzymes cystathionine β-synthase (CBS) or cystathionine γ-lyase (CSE), and has been shown to modulate p38 MAPK activity and contribute to tumor cell proliferation (24, 52). Inhibition of H_2_S production effectively reverses these effects, resulting in reduced tumor growth, increased radiosensitivity, and downregulated EMT-associated protein expression, such as Snail and N-cadherin (24). These findings suggest that H_2_S-driven activation of p38 MAPK is a crucial mechanism of radiation-induced aggressiveness, and targeting H_2_S metabolism may potentially enhance radiosensitivity.

Moreover, p38 MAPK can exerts its pro-tumorigenic effects through its downstream effectors such as MAPK-activated protein kinase 2 (MK2), which is known to promote the phosphorylation of heat shock protein 27 (HSP27) (53). This modification subsequently activates the NF-κB signaling cascade, leading to increased expression of pro-inflammatory cytokines, including tumor necrosis factor-alpha (TNF-α), interleukin-1 alpha (IL-1α), interleukin-1 beta (IL-1β), and interleukin-6 (IL-6), thereby facilitating tumor progression (53). Berggren et al. (2019) demonstrated that MK2 inhibition following RT significantly reduced cytokine induction and delayed tumor regrowth in a murine model of head and neck squamous cell carcinoma (HNSCC), positioning MK2 as a potential therapeutic target to mitigate radiation-induced metastasis (53).

Interestingly, even low-dose radiation can activate p38 MAPK pathway, which in turn facilitates tumor migration (25). For instance, in glioma, lung, and cervical cancer cell lines, exposure to low-dose γ-radiation (10–20 cGy) selectively activates p38 MAPK-mediated invasion via the upregulation of connexin-43 (Cx43), a gap junction protein (25). Notably, knockdown of Cx43 effectively prevents radiation-induced p38 MAPK phosphorylation and suppresses the associated cell migration and invasion, whereas it does not affect ERK1/2 activity or cell proliferation (25). Although the precise mechanism of their direct interaction is not fully understood, Cx43 may promote p38 MAPK signaling by forming hemichannels that release pro-migratory factors (54). These findings suggest that targeting Cx43 could be a potential strategy to limit invasion induced by low-dose radiation.

**Figure 3:**
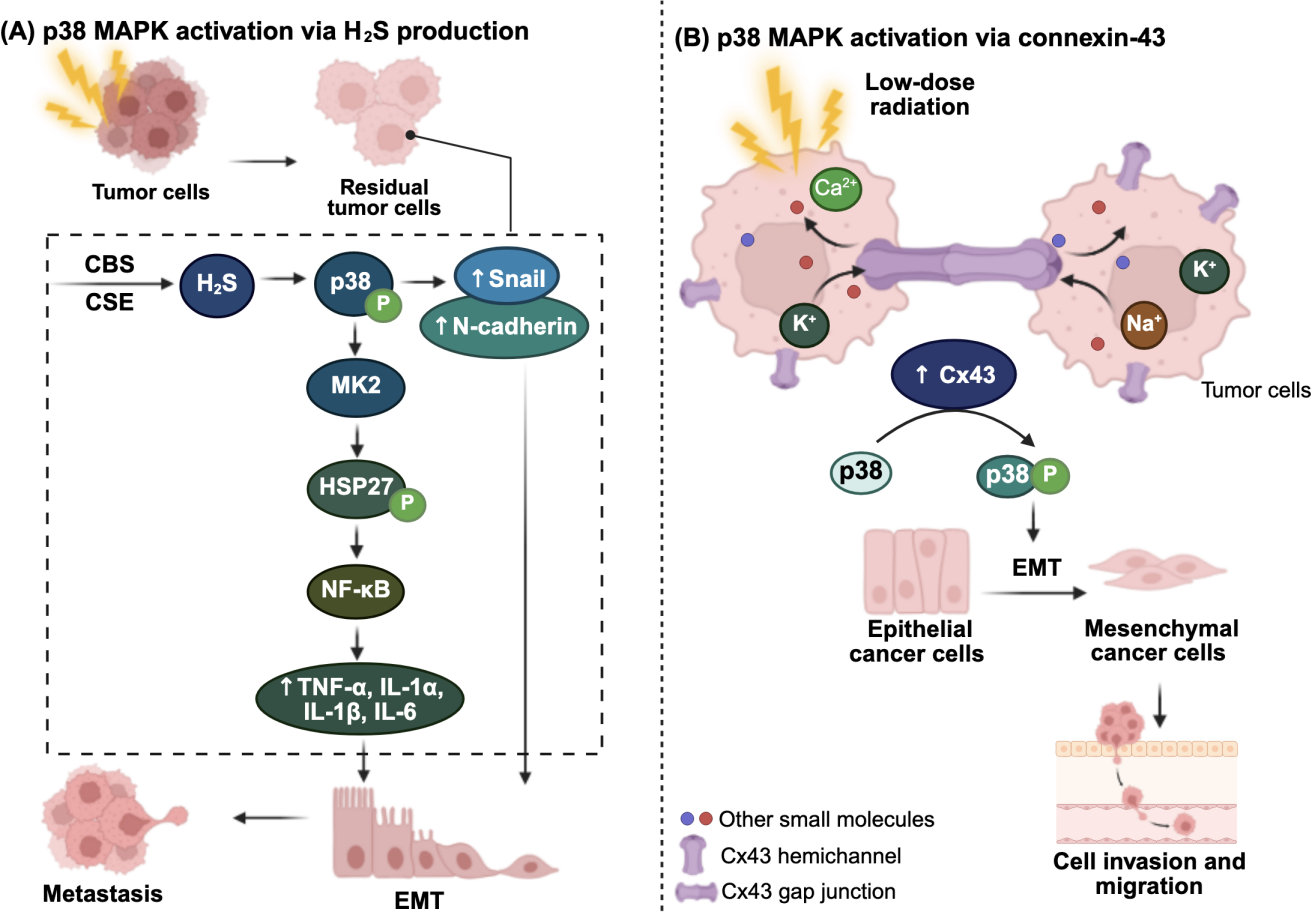
Radiation-induced inflammatory p38 MAPK activation via **(A)** hydrogen sulfide (H_2_S) and **(B)** connexin-43 (Cx43). (A) In residual tumor cells, endogenous H2S production is constitutively upregulated through increased expression of the two key H2S-producing enzymes, cystathionine-γ-lyase (CSE) and cystathionine-β-synthase (CBS). Elevated H_2_S levels activate and phosphorylate p38 mitogen-activated protein kinase (MAPK), which promotes epithelial-mesenchymal transition (EMT) by upregulating EMT markers, such as Snail and N-cadherin. Activated p38 also stimulates MAPK-activated protein kinase 2 (MK2; MAPKAPK2), which phosphorylates heat shock protein 27 (HSP27), ultimately leading to NF-κB activation. This results in increased secretion of immune modulating inflammatory cytokines, further driving tumor progression and metastasis. **(B)** Low-dose γ-radiation induces p38 MAPK activation through upregulation of Cx43. Cx43 forms hemichannel between neighboring tumor cells, facilitating the release of signaling molecules or pro-migratory factors, thereby promoting EMT and enhancing cell invasion and migration. The figure was created using BioRender (https://BioRender.com).

Overall, both high-dose and low-dose irradiation converge on the p38 MAPK pathway through distinct but interconnected mechanisms, including H_2_S signaling, MK2-driven inflammation, and Cx43-mediated invasion, to promote tumor aggressiveness. These pathways highlight multiple therapeutic opportunities to enhance radiosensitivity and inhibit radiation-induced metastasis. **Figure 3** illustrates the mechanisms by which irradiation activates the p38 MAPK pathway to promote cancer metastasis.

### 2.4 Radiation-induced inflammatory NF-κB signaling

NF-κB is a family of inducible transcription factors that play a pivotal role in regulating genes involved in inflammation, innate and adaptive immunity, cell proliferation, apoptosis, and oncogenesis. The NF-κB family comprises five structurally related members: RelA (p65), RelB, c-Rel, NF-κB1 (p50), and NF-κB2 (p52) (55). In unstimulated cells, NF-κB dimers, especially p50/RelA are sequestered in the cytoplasm by NF-κB protein inhibitors (IκB) (55). Upon stimulation by pathogen-associated molecular patterns (PAMPs) and damage-associated molecular patterns (DAMPs), such as TNF-α, ROS, or IR, the IκB kinase (IKK) complex becomes activated and triggers IκB degradation (55). This releases NF-κB, allowing its translocation into the nucleus to activate transcription of target genes (55). However, dysregulated or constitutive activation of NF-κB can lead to chronic inflammation and uncontrolled immune modulation, which drives cancer initiation and progression(56).

Notably, IR-induced inflammatory NF-κB activation has been linked to cancer metastasis in various tumor types (26–28, 56, 57). For instance, irradiated T cell-derived exosomes promoted esophageal cancer cells metastasis by upregulating β-catenin and the NF-κB/Snail pathway, facilitating EMT (26). In lung cancer, IR has been shown to promote tumor cell invasion and migration through the upregulation of IL-1β and its receptor, interleukin-1 receptor type I (IL-1RI) or II (IL-1RII) (27). Although IL-1RII is traditionally viewed as a decoy receptor that competes with IL-1RI to bind IL-1β and prevent NF-κB activation, Liu et al. (2022) demonstrated that IL-1RII can also promote oncogenic signaling by activating the Janus kinase 2 (JAK2)/signal transducer and activator of transcription 3 (STAT3) pathway in clear cell renal cell carcinoma (ccRCC) (58, 59). In HCC, activation of JAK2/STAT3 has been shown to increase matrix metalloproteinase 2 (MMP2), MMP9, and vascular endothelial growth factor (VEGF), promoting cell migration and invasion (60).

Furthermore, siRNA-mediated silencing and pharmacological inhibition of receptor-interacting protein kinase 1 (RIP1) and NF-κB significantly reduced IL-1β secretion, while direct inhibition of IL-1β suppressed IR-induced EMT and cell invasion (27). These findings suggest that IR may concurrently activate NF-κB to promote IL-1β release, thereby enhancing metastasis through EMT (27). Similarly, low-dose radiation (4 Gy) activates NF-κB signaling through the upregulation of chemokine (C-X-C motif) ligand 1 (CXCL1) expression in NSCLC, facilitating EMT and invasion (28). In addition, NF-κB also mediates IR-induced EMT through the RIP1/proto-oncogene tyrosine-protein kinase Src (Src kinase)/STAT3 pathway, which enhances the activity of ECM-degrading enzymes, such as MMP-2 and MMP-9 (56). Targeting these pathways with inhibitors that suppress NF-κB activity and MMP-9 expression may demonstrate promising immune modulation that could result in inhibiting IR-induced metastasis (57).

**Figure 4:**
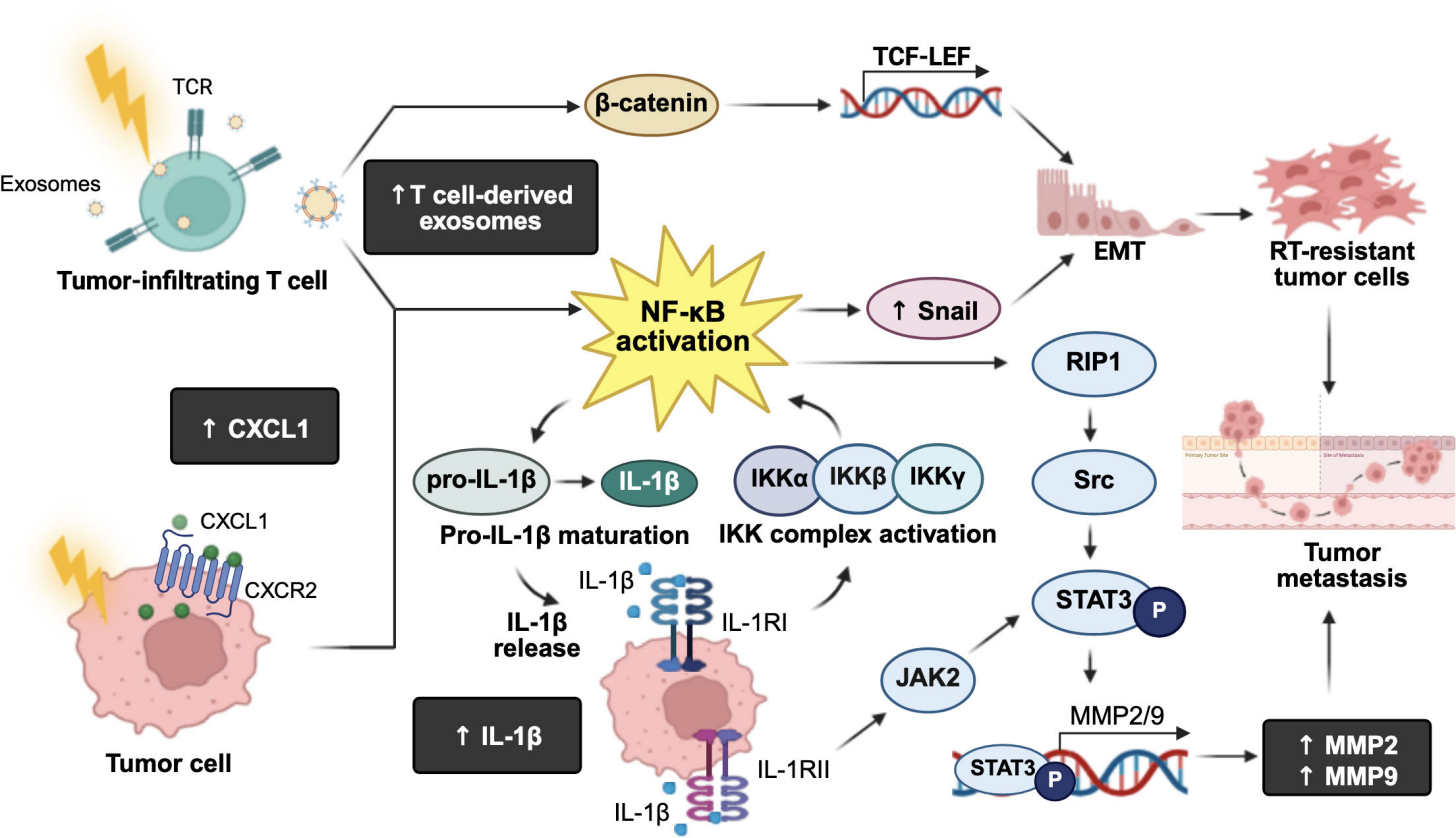
Roles of radiation-induced NF-κB immune modulation leading to cancer metastasis. Exposure to ionizing radiation activates the IκB kinase (IKK) complex, which phosphorylates inhibitor of NF-κB (IκB) proteins, leading to their proteasomal degradation and the subsequent activation of NF-κB. Activated NF-κB triggers the maturation and production of interleukin-1β (IL-1β), and its receptors, IL-1 receptor type I (IL-1RI) and type II (IL-1RII). IL-1RI further activates the IKK complex, creating a positive feedback loop that amplifies NF-κB signaling. On the other hand, IL-1RII activates the Janus kinase 2 (JAK)/signal transducer and activator of transcription 3 (STAT3) pathway, leading to the transcription of matrix metalloproteinase (MMP)-2 and MMP9, which promote tumor metastasis. Additionally, activated NF-κB upregulates MMP2/9 via the receptor-interacting protein kinase 1 (RIP1)/proto-oncogene tyrosine-protein kinase Src/STAT3 signaling pathway, further enhancing tumor metastatic potential. Irradiated tumor-infiltrating T cells-derived exosomes can upregulate β-catenin and NF-κB, inducing Snail expression and transcriptional activation of T cell factor-lymphoid enhancer factor proteins (TCF-LEF) target genes. This results in an upregulation of epithelial-mesenchymal transition (EMT), supporting radioresistant tumor cell survival. Moreover, low-dose radiation increases chemokine C-X-C motif ligand 1 (CXCL1) expression and activates NF-κB signaling to promote EMT, contributing to tumor invasion and metastasis. The figure was created using BioRender (https://BioRender.com).

Collectively, these findings clearly demonstrate the role NF-κB as a central player in radiation-induced metastasis, promoting immune overactivation, EMT, migration, and invasion through multiple interconnected signaling cascades (**Figure 4**). These insights underline the importance of developing targeted therapies to mitigate the pro-metastatic effects of RT.

## 3 Drug repurposing and clinical trial studies on radiation-induced inflammation in cancer metastasis

While RT remains a cornerstone of cancer management due to its effectiveness in reducing primary tumor burden, it often falls short in preventing post-treatment metastatic progression (17). In parallel, tumor cells can detect radiation-induced DNA damage and activate a cascade of immune signaling pathways to repair the damage, protecting them from therapy-induced cytotoxicity (38). Consequently, strategies have been developed to interfere with DDR pathways in tumor cells, and accumulating evidence suggests that the combination of RT with target agents may enhance the therapeutic efficacy and tumor control. **Table 1** provides an overview of FAK, PI3K/AKT pathway, p38 MAPK, and NF-κB inhibitors that have been evaluated in preclinical studies and phase I/II clinical trials for their potential to improve outcomes when used in combination with RT (53, 61–80). However, acquired resistance to these targeted therapies may develop over time, and the risk of excessive adverse events associated with combination regimens remains a concern (81). In light of this, drug repurposing—a strategy that identifies new applications for existing therapeutics—offers a cost-effective and expedited approach to enhance the efficacy of RT (82). By leveraging known pharmacodynamics and safety profiles, repurposed drugs may serve as promising candidates to target key molecular pathways involved in IR-induced metastasis (**Table 2**) (82). **Table 2** summarizes repurposed drugs with potential activity as FAK, PI3K/AKT pathway, p38 MAPK, and NF-κB inhibitors in cancer treatment. (84–100).

**Table 1:** Drugs combined with radiotherapy for cancer treatment.

Types of inhibitors	Compound	Target cancer	Biomolecule targets	Functions	Phase	Treatment	Reference
FAK inhibitors	VS-4718	PDAC	FAK	Reduces stromal density (fibrosis) in cancer by targeting cancer-associated fibroblasts. Modulates the TME to enhance immune infiltration and sensitivity to RT. Enhances immune response.	*In vivo*	Simultaneous administration of the drug compound and irradiation	(61)
	Ifebemtinib (IN10018)	PDAC	FAK	Acts as a radiosensitizer. Promotes immune modulation by increasing CD8^+^ T cell infiltration in tumors. Reduces immunosuppressive cells within the TME.	*In vivo*	Postoperative adjuvant treatment	(62)
	Defactinib			Resensitizes RT resistance PDAC. Modulate DNA damage repair/responses, ROS pathways, cell cycle regulation, and inflammatory signaling. Induce immune priming.	*In vivo* and clinical trial (NCT04331041)	Concomitant therapy	(63)
	TAE226	Glioblastoma multiforme	FAK and IGF-IR	Induces cytotoxicity, reduces clonogenic survival, and sphere formation in glioblastoma cells. Radiosensitizes specific glioblastoma cell lines when combined with X-rays. Induces apoptosis but does not significantly trigger autophagic cell death.	*In vitro*	Pre-irradiation treatment	(64)
PI3K/AKT pathway inhibitors	Omipalisib (GSK2126458)	NPC	PI3K isoforms: p110α, p110β, p110γ, and p110δ mTOR complexes: mTORC1 and mTORC2	Inhibitors of cell proliferation, migration, and invasion. Sensitizers to IR by increasing DNA damage, enhancing G2–M phase cell cycle delay, and inducing apoptosis.	*In vivo*	Pre-irradiation administration	(65)
	Gedatolisib (PKI-587)		PI3K isoforms: p110α and p110γ mTOR complexes: mTORC1 and mTORC2				
	BEZ235	Prostate cancer	PI3K isoforms: p110α, p110β, p110γ, and p110δ mTOR complexes: mTORC1 and mTORC2	Acts as a radiosensitizer. Induces G2/M cell cycle arrest. Increases residual DNA damage post-irradiation. Inhibits DNA repair processes. Sensitizes tumor cells under both normoxic and hypoxic conditions.	*In vitro* and *in vivo*	Pre-irradiation treatment	(66)
	Nelfinavir	HNSCC and lung carcinoma	Targets VEGF, HIF-1α, and Sp1 (a transcription factor regulating VEGF expression)	Inhibits the PI3K/AKT signaling pathway. Downregulates VEGF and HIF-1α, reducing angiogenesis and improving tumor oxygenation. A radiosensitizer by improving oxygenation.	*In vitro* and *in vivo*	Pre-irradiation treatment	(67)
		Advanced rectal cancer	PI3K and AKT	Act as a radiosensitizer.	Phase I clinical trial (EudraCT number: 2010-020621-40)	Neoadjuvant and concomitant	(68)
		Locally advanced pancreatic cancer	Protease inhibitor	Act as a radiosensitizer.	Phase I and Phase II clinical trial (ISRCTN50083238)	Concomitant with capecitabine-based chemoradiotherapy	(69)
	Buparlisib (BKM120)	OSCC	PI3K	Acts as a radiosensitizer.	*In vitro*	Pre-irradiation treatment	(70)
		Glioblastoma		Acts as a radiosensitizer. Enhancing the activity of the combination drug, temozolomide.	Phase I clinical trial (NCT01473901)	Cohort I: Buparlisib was taken as adjuvant drug. Cohort II: Buparlisib was taken concomitantly with RT and temozolomide	(71)
	Alpelisib (BYL719)	OSCC	PI3Kα	Acts as radiosensitizer.	*In vitro*	Pre-irradiation treatment	(70)
		Locoregionally advanced HNSCC			Phase I clinical trial (NCT02537223)	Concomitant chemoradiotherapy	(72)
		Locally advanced stage III-IVB HNSCC			Phase 1b clinical trial (NCT02282371)	Concomitant cetuximab-based RT	(73)
p38 MAPK pathway inhibitors	Adezmapi-mod (SB203580)	Breast cancer	p38 MAPK	Inhibits the activation of p38 MAPK. When combined with IMRT, it synergistically inhibits the proliferation of cancer cells.	*In vitro*	Post-irradiation treatment	(74)
	Ralimetinib (LY2228820)	Glioblastoma	p38 MAPK inhibitor	Inhibits the p38 MAPK pathway by targeting MK2, a key downstream effector involved in inflammation, cell survival, and stress responses.	Phase I clinical trial (NCT02364206)	Concomitant chemoradiotherapy	(75)
	PF-3644022	HNSCC	MK2	Suppresses radiation-induced MK2 phosphorylation. Reduces levels of phosphorylated HSP27, pro-inflammatory cytokines, and EMT gene expression (ie. SNAI1, SNAI2, VIM). In patient-derived xenograft combined treatment with PF-3644022 with RT led to improved survival.	*In vitro* and *in vivo*	Administer concurrently with RT	(53)
NF-κB inhibitors	Dehydroxy-methyl-epoxy-quinomicin (DHMEQ)	Prostate cancer	NF-κB	Inhibits radiation-induced NF-κB activation. Enhances radiosensitivity. Induces G2/M cell cycle arrest. Increase apoptosis of cancer. In combination with radiation, it significantly reduces tumor growth and colony formation.	*In vitro* and *in vivo*	*In vitro*: pre-irradiation treatment *In vivo*: Concurrent administration	(76)
	Fimepinostat (CUDC-907)	Pediatric high-grade gliomas	NF-κB and FOXM1	Acts as a radiosensitizer. Impairs DDR pathways (homologous recombination and non-homologous end joining). Induces G1 cell cycle arrest. Enhance radiation-induced DNA damage and apoptosis. Reduces the transcriptional activity of NF-κB and FOXM1, critical for DNA repair.	*In vitro*	Pre-irradiation treatment	(77)
	Dimethyl-amino-parthenolide (DMAPT)	NSCLC	NF-κB	Inhibits NF-κB activation and signaling pathways. Blocks DNA double-strand break repair by impairing homologous recombination and non-homologous end joining. Enhancing the persistence of DNA damage post-radiation and increasing apoptosis.	*In vitro* and *in vivo*	Pre-irradiated treatment	(78)
		Prostate cancer	NF-κB, and apoptosis signaling	Radiosensitizes tumor tissue. Protects normal tissue from radiation-induced apoptosis.	*In vivo*	Pretreatment with DMAPT before radiation	(79)
	Nafamostat mesilate (NM)	Gallbladder cancer	NF-κB	Inhibits radiation-induced activation of NF-κB. Promotes apoptosis, G2/M cell cycle arrest, and reduces cancer cell migration and invasion. Improves radiosensitivity *in vitro* and *in vivo* models.	*In vitro* and *in vivo*	Pre-irradiated treatment	(80)

FAK, focal adhesion kinase; PDAC, pancreatic ductal adenocarcinoma; TME, tumor microenvironment; RT, radiotherapy; CD8^+^, cluster of differentiation 8-positive; ROS, reactive oxygen species; IGF-IR, insulin-like growth factor-I receptor; NPC, nasopharyngeal carcinoma; mTOR, mechanistic target of rapamycin; mTORC1, mechanistic target of rapamycin complex 1; mTORC2, mechanistic target of rapamycin complex 2; IR, ionizing radiation; HNSCC, head and neck squamous cell carcinoma; VEGF, vascular endothelial growth factor; HIF-1α, hypoxia-inducible factor 1 alpha; Sp1, specificity protein 1; OSCC, oral squamous cell carcinoma; PI3K, phosphoinositide 3-kinases; AKT, protein kinase B; p38 MAPK, p38 mitogen-activated protein kinase; IMRT, intensity-modulated radiotherapy; MK2, MAPK-activated protein kinase 2; HSP27, heat shock protein 27; EMT, epithelial–mesenchymal transition; SNAI1, Snail family transcriptional repressor 1; SNAI2, Snail family transcriptional repressor 2 (Slug); VIM, vimentin; NF-κB, nuclear factor-kappa B; FOXM1, forkhead box protein M1; DDR, DNA damage repair; NSCLC, non-small-cell lung cancers

**Table 2:** List of repurposed drugs for cancer treatment evaluated in preclinical studies.

Types of inhibitors	Drug	Primary indication	Functions	Cancer Type	References
FAK inhibitors	Amprenavir	HIV infection	Induces apoptosis by inhibiting ERK2 kinase activity. Reduces tumor proliferation.	Merlin-negative tumors	(83)
	Bosutinib	Chronic myeloid leukaemia	Inhibits FAK and Src kinases. Reduces tumor cell proliferation and induces apoptosis. Inhibits EGFR and Src/NF-κB/survivin signaling pathways.	Breast cancer, HNSCC, cervical cancer	(83)
	Ferric Derisomaltose	Iron deficiency anemia	Inhibits FAK through interactions with Cys502, Asp564 Acts as a radiosensitizer.	–	(83)
	Cerivastatin	Hypercholesterolemia	Inhibits FAK phosphorylation	Glioblastoma	(84)
			Inhibits RhoA prenylation	Breast cancer	(85)
PI3K/AKT pathway inhibitors	Promethazine	Antihistamine, antiemetic, and sedative	Induces autophagy-associated apoptosis in cancer cells. Inhibits the PI3K/AKT/mTOR pathway by reducing p-PI3K expression. Increases ROS production, leading to AMPK activation, upregulation of ULK1, and phosphorylation of Beclin-1, thereby promoting autophagy.	Chronic myeloid leukemia	(86)
			Downregulate PI3K/AKT signaling cascade Downregulate Bcl-2 expression and activate caspase-3 Promotes apoptosis.	Colorectal cancer	(87)
	Itraconazole	Antifungal	Inhibits cancer cell proliferation and induces apoptosis Downregulates Wnt growth factor and β-catenin, increasing the expression of Axin-1. Reduces phosphorylation of PI3K, AKT, and mTOR, inhibiting protein synthesis and tumor cell growth. Downregulates Shh and Smo proteins. Increases ROS levels, promoting apoptosis. Activates caspase-8 and downstream caspase-3 promotes cell death.	HCC	(88)
	Nelfinavir	HIV-1 protease inhibitor	Induces endoplasmic reticulum stress, unfolded protein response, and activates pro-apoptotic pathways via PERK phosphorylation and CHOP expression (7, 8). Inhibits phosphorylation of key signaling molecules like AKT, STAT3, and ERK1/2, leading to apoptosis (7, 8).	Multiple myeloma, NSCLC and breast cancer	(89, 90)
	Sevoflurane	Anaesthesia	Inhibits proliferation, migration, and invasion of glioma cells while promoting apoptosis. Inhibits the PI3K/AKT signaling pathway, promotes proliferation, and induces cell apoptosis.	Glioma	(91)
	Isoflurane	Anaesthesia	Isoflurane inhibits growth, migration, and invasion of hepatic carcinoma cells. It promotes apoptosis. Regulates aggressiveness of cancer cells via the PI3K/AKT-mediated NF-κB signaling pathway by suppresses PI3K and AKT expression. Downregulates NF-κB activity, reducing inflammation and tumor progression.	HCC	(92)
p38 MAPK activators	Atorvastatin	Treat hyperlipidemia and reduce cardiovascular risk	Blocks HMGCR Disrupting critical cellular functions in cancer, i.e., membrane integrity, cell cycle progression, and signal transduction. Induces apoptosis and autophagy. Causes cell cycle arrest at the G1 Inhibits adhesion, migration, invasion, and angiogenesis. Downregulates VEGF and MMP9 to prevent metastasis and tumor vascularization. Affects multiple signaling pathways, including AKT/mTOR, MAPK, and Myc.	Ovarian cancer	(93)
	Lovastatin		Activates LKB1-AMPK-p38 MAPK-p53-survivin cascade to induce apoptosis. Modulates p53 phosphorylation and acetylation, enhancing tumor suppressor activity.	Breast cancer	(94)
	Fluvastatin		Induce apoptosis via increased DNA fragmentation Activation of caspase-3, PARP, and Bax Suppression of anti-apoptotic Bcl-2	Malignant lymphoma	(95)
	Simvastatin				
NF-κB inhibitors	Disulfiram and copper (DS/Cu)	Anti-alcoholism drug	DS/Cu complex induces cytotoxicity in lymphoid malignant cells (14). Induces apoptosis in leukemia cells by: Generating ROS (14, 15). Activating JNK (14). Inhibiting the pro-survival pathways NF-κB and Nrf2 (14).	Lymphoid malignancies and acute myeloid leukemia	(96, 97)
	Niclosamide	Anthelmintic	Inhibits the NF-κB pathway, Blocks tumor necrosis factor-induced IκBα phosphorylation, translocation of p65, and expression of NF-κB-genes. Increases the levels of ROS in AML cells, contributing to apoptosis	Acute myelogenous leukemia	(98)
	Celecoxib	Non-steroidal anti-inflammatory drug for pain relief and inflammation reduction	Suppresses the proliferation and metastasis of pancreatic cancer cells by down-regulating the activities of STAT3, NF-κB, and L1CAM.	Pancreatic cancer	(99)

### 3.1 Targeting FAK to remodel the TME and enhance radiosensitivity

As discussed earlier, IR-induced focal adhesion kinase (FAK) activation initiates a cascade of intracellular signaling pathways that promote cell survival, migration, and proliferation (34, 100). Given FAK’s central role in these oncogenic pathways, it has emerged as a promising therapeutic target for overcoming radiation-induced cancer progression.

One of the most notable roles of FAK inhibitors is their ability to modulate the TME by altering the composition and behavior of immune and stromal cells within the TME (61). For instance, in the pancreatic cancer mouse model, FAK inhibition by VS-4718 in combination with low-dose radiation (10 Gy) has been shown to reduce stromal density and tumor hypoxia, thereby facilitating immune cell infiltration and enhancing the radiosensitivity of tumor cells (61). Similarly, Osipov and coworkers demonstrated that the adjuvant use of ifebemtinib (IN10018), a FAK inhibitor, improved survival in RT-treated murine pancreatic ductal adenocarcinoma (PDAC) model in a cluster of differentiation 8-positive (CD8^+^) T cell-dependent manner (62). Although the combination regimen increased the proportions of tumor-associated macrophages (TAMs) and regulatory T cells (Tregs), these immunosuppressive effects were offset by a robust infiltration of CD8^+^ T cells (62). These findings suggest that targeting FAK may potentially enhance the efficacy of RT by modulating the TME to favor antitumor immune cell infiltration (62). TAE226 (2-(5-chloro-2-(2-methoxy-4-(4-morpholinyl)phenylamino)pyrimidin-4-ylamino)-N-methylbenzamide), a potent adenosine triphosphate (ATP)-competitive FAK inhibitor, has also demonstrated significant antiproliferative and antitumor effects in glioblastoma cells, markedly reducing cell survival in a concentration- and time-dependent manner (64) (64).However, TAE226 exhibited minimal to no effect on enhancing radiosensitivity, suggesting that its efficacy may be tumor type-dependent and highlighting the importance of selecting inhibitors based on tumor-specific biology (64). Despite these promising preclinical findings, no FAK inhibitors have yet received clinical approval, and their efficacy in combination with RT remains to be fully elucidated. A phase II clinical study (NCT04331041) is ongoing to evaluate the efficacy and safety profile of combining FAK inhibitor defacitinib and SBRT in patients with advanced PDAC (63).

Meanwhile, several repurposed drugs have shown potential in inhibiting FAK-mediated signaling pathways and are currently in early-stage preclinical evaluation. For instance, an *in silico* study by Siyah (2024) identified amprenavir, originally approved for Human Immunodeficiency Virus (HIV) treatment; ferric derisomaltose, used for iron deficiency anemia; and bosutinib, a tyrosine kinase inhibitor, as compounds with high predicted binding affinity for the FAK protein (83). These findings suggest that these agents may directly bind to FAK and effectively inhibit the activation of its downstream oncogenic signaling pathways (83). Cerivastatin, a statin commonly used to lower cholesterol, has been shown to impair actin stress fiber formation and inhibit focal adhesion assembly by downregulating FAK phosphorylation (84). This results in the loss of cell shape and tension, detachment from the ECM, and ultimately reduces migration and invasion of glioblastoma cells (84). In breast tumor cells, cerivastatin exerts its anticancer effects by inhibiting RhoA prenylation, thereby suppressing the RhoA/ROCK pathway and inducing cytoskeletal destabilization, which leads to a loss of traction forces essential for cell migration (85). Additionally, it disrupts the RhoA/FAK/AKT signaling pathway, which regulates gene transcription involved in proliferation and invasion (85). Downstream pathways involving NF-κB, β-catenin, and activator protein-1 (AP-1) are also further suppressed by cerivastatin, collectively contributing to reduced tumor cell proliferation, invasion, and survival (85). These findings suggest that, beyond direct FAK inhibition, agents like statins can indirectly target FAK-related signaling to reduce tumor invasiveness and potentially improve radiosensitivity, expanding therapeutic options.

Taken together, these findings underscore the promising potential of targeting the FAK signaling cascade to counteract metastasis and enhance radiosensitivity. Moving forward, the integration of FAK inhibitors with RT warrants rigorous clinical validation through well-designed trials to confirm their therapeutic benefit while minimizing toxicity and adverse effects (11).

### 3.2 Targeting PI3K/AKT pathway for radiosensitization

Dysregulation of the PI3K/AKT pathway in cancer cells is well known to contribute to drug and treatment resistance (101). Given the differential activation of this pathway in tumor cells compared to normal cells, targeting it has been proposed as a promising strategy to enhance tumor cell sensitivity and improve therapeutic efficacy (101). As such, investigating inhibitors of the PI3K/AKT pathway represents a worthwhile strategy to overcome treatment resistance in cancer 102. 

To date, multiple ongoing clinical trials are evaluating the safety and efficacy of drugs targeting the PI3K/mechanistic target of rapamycin (mTOR) pathways (66, 71, 73, 102). Emerging evidence suggests that combining these agents with RT enhances antitumor efficacy (66, 71, 73, 102). For instance, dual PI3K/mTOR inhibitors such as omipalisib (GSK2126458/GSK458), gedatolisib (PKI-587), and dactolisib (BEZ235) have been shown to effectively suppress the phosphorylation of mTOR, AKT, and eukaryotic translation initiation factor 4E-binding protein 1 (4EBP1) in NPC and prostate cancer cells. This suppression leads to improved antitumor activity and radiosensitization, primarily through inhibition of DNA repair, induction of G2/M cell cycle arrest, and increased apoptosis (66, 67).

In a phase I clinical study (NCT00972686), omipalisib demonstrated tolerability at a dose of 2.5 mg/day in patients with breast, renal cell, bladder, or endometrial cancers, without any life-threatening adverse events (102). However, durable objective responses were observed in only 5% of patients, with two patients achieving sustained responses lasting over four years. Notably, *PIK3CA* expression did not correlate with clinical outcomes in this study (102) (102).In contrast, a phase Ib clinical trial (NCT02282371) evaluating alpelisib (BYL719), an oral class I α-specific PI3K inhibitor, in combination with cetuximab and IMRT, reported promising outcomes in patients with locally advanced HNSCC, particularly among those harboring *PIK3CA* mutations (73). This differential clinical efficacy may be attributed to the distinct targeting profiles of the two agents. Alpelisib primarily inhibits both the wild-type and mutant PI3Kα isoforms, whereas omipalisib targets a broader range of PI3K isoforms, including mutant forms of *PIK3CA* and mTOR complexes (73, 102). Despite this broader targeting capacity, omipalisib has been shown to primarily target the mTOR signaling pathway, with its downstream proteins being significantly downregulated in a dose-dependent manner (103, 104). The findings suggest that distinct mechanisms of action of PI3K inhibitors may contribute to variations in clinical efficacy across diverse cancer types. Nonetheless, these results highlight the urgent need for more reliable biomarkers to identify patients who are likely to respond to PI3K inhibitors, in order to improve therapeutic efficacy and clinical outcomes.

Besides that, buparlisib (BKM120), a pan-class I PI3K inhibitor, has demonstrated synergistic effects with radiation and mTOR inhibitors in preclinical models of oral squamous cell carcinoma (OSCC) (70). However, its clinical application has been hindered by toxicity (70, 71). In a phase I study (NCT01473901) evaluating buparlisib in combination with temozolomide and RT in patients with newly diagnosed glioblastoma, the trial was terminated early due to the inability to determine the maximum tolerated dose, coupled with a high incidence of adverse events (71). This, in turn, raises concerns about the viability of buparlisib for future development 72.

Given the limitations of current targeted therapies, several non-oncogenic drugs, such as promethazine, itraconazole, and nelfinavir, have shown promising anticancer activity by targeting the PI3K signaling pathway. For instance, promethazine, a first-generation antihistamine with sedative and antiemetic properties, has been shown to inhibit the PI3K/AKT/mTOR signaling in chronic myeloid leukemia by downregulating phosphorylated PI3K (p-PI3K) (86). This relieves autophagy suppression and activates AMP-activated protein kinase (AMPK), which further inhibits mTOR and downregulates the anti-apoptotic protein myeloid cell leukemia 1 (MCL-1), thereby promoting autophagy. (86) Similarly, in colorectal cancer models, promethazine has been shown to reduce p-PI3K and phosphorylated AKT (p-AKT) levels, suppress cell proliferation, and induce apoptosis by downregulating B-cell lymphoma 2 (Bcl-2) and activating caspase-3 (87). Itraconazole, an antifungal agent, suppresses HCC cell growth and induces apoptosis via multiple mechanisms, including inhibition of the Hedgehog, Wnt/β-catenin, and AKT/mTOR/ribosomal protein S6 kinase (S6K) signaling pathways, as well as by inducing ROS production and activating death receptor signaling 89.

Notably, nelfinavir, originally developed as an HIV protease inhibitor, has been extensively studied for its antitumor activity due to its ability to inhibit the PI3K/AKT/mTOR signaling pathways and function as a radiosensitizer (105, 106). In multiple myeloma, nelfinavir induces apoptotic cell death by activating caspase 3 and downregulating the phosphorylation of AKT, STAT3, and ERK1/2, which leads to activation of the pro-apoptotic unfolded protein response pathway (89). Beyond its pro-apoptotic effects, nelfinavir also modulates the TME by downregulating hypoxia-inducible factor 1 alpha (HIF-1α) and VEGF, thereby improving tumor oxygenation and suppressing angiogenesis (67). This effect is mediated through inhibition of AKT signaling and reduced specificity protein 1 (Sp1) phosphorylation (67). The therapeutic efficacy of nelfinavir has been evaluated in a non-randomized, open-label clinical trial, SONATINA (European Union Drug Regulating Authorities Clinical Trial Database, EudraCT number: 2010-020621-40), in combination with RT in patients with advanced rectal cancer (68). This combination regimen was well tolerated and associated with increased tumor perfusion (68). Notably, the median tumor cell density decreased from 24.3% at baseline to 9.2% seven days post-treatment, supporting its potential as a radiosensitizer for targeting metastatic disease (68). However, the therapeutic efficacy of nelfinavir appears to be cancer-type dependent. Despite evidence that nelfinavir induces AKT inhibition in both *in vitro* and *in vivo* models of prostate cancer, it did not lead to improvements in local tumor control and radiosensitivity (107). Similarly, in the multi-center, two-stage SCALOP-2 trial (ISRCTN50083238), the combination of nelfinavir with capecitabine-based chemoradiotherapy was well tolerated in patients with locally advanced pancreatic cancer, but it failed to improve progression-free survival (PFS) (69, 108). These findings suggest that tumor types may potentially play critical roles in shaping the cytotoxicity of nelfinavir, underscoring the need for cancer-specific therapeutic strategies and more reliable predictive biomarkers.

Overall, clinical outcomes indicate that PI3K/AKT pathway inhibitors may benefit only a subset of patients whose tumors are highly dependent on this signaling axis. Despite encouraging preclinical results, their translation into clinical success has been limited, primarily due to challenges in patient stratification and the management of treatment-related toxicities. Continued investigation is warranted, particularly in identifying robust predictive biomarkers, such as PNUTS overexpression and PTEN loss, which could help refine patient selection and enhance the therapeutic efficacy of PI3K/AKT/mTOR-targeted therapies (109).

### 3.3 p38 MAPK pathway in therapy implications

Activation of p38 MAPK in response to stressors such as IR has been implicated in promoting metastasis by driving inflammation, EMT, and increased cellular motility (110). Therefore, targeting p38 MAPK represents a promising strategy for radiosensitization. Selective p38 inhibitors such as adezmapimod (SB203580) and ralimetinib (LY2228820) have demonstrated synergistic effects with RT in preclinical and early clinical studies by enhancing apoptosis and impairing DNA repair mechanisms (74, 75). For instance, Zhao et al. (2018) showed that adezmapimod (SB203580), a selective p38α and p38β MAPK inhibitor, enhanced tumor suppression and reduced proliferation in MCF-7 breast cancer cells when combined with RT (74). Similarly, encouraging results were reported in a phase I clinical trial evaluating ralimetinib (LY2228820), a selective, ATP-competitive p38 MAPK inhibitor, in combination with standard chemoradiotherapy (temozolomide + RT) for glioblastoma patients (75). The trial showed a median PFS of 12.8 months and an overall survival (OS) of 24.5 months, outperforming historical benchmarks (75). These early-phase clinical trial results illustrated the therapeutic potential of p38 MAPK inhibition for radiosensitization. Nevertheless, larger-scale studies and biomarker-driven trials are needed to determine whether p38 inhibition can be effectively integrated into standard cancer therapies.

Despite numerous p38 MAPK inhibitors being investigated, none have received regulatory approval for clinical use (98). This is largely due to the pathway multifaceted roles, limited sustained efficacy, and significant adverse effects such as hepatotoxicity, cardiotoxicity, and tachyphylaxis, all of which collectively complicate the assessment of their therapeutic benefits in cancer treatment (111). To address these limitations, statins have emerged as potential modulators of the p38 MAPK pathway. Statins such as atorvastatin, lovastatin, fluvastatin, and simvastatin have been shown to induce apoptosis and G1 cell cycle arrest in ovarian, breast, and lymphoma cells via AKT/mTOR inhibition and p38 MAPK activation (93–95). However, this mechanism appears to conflict with the strategies proposed by Zhao et al. (2018) and Biau et al. (2021), where p38 MAPK inhibition suppressed IR-induced tumor metastasis (74, 75). These contrasting findings suggest that p38 MAPK may act as either a tumor suppressor or promoter, depending on the cancer type and TME, further complicating its utility as a therapeutic target in oncology.

Given these complexities, recent cancer research has shifted toward targeting MK2, the downstream effector of p38 MAPK (111). MK2 is a kinase involved primarily in regulating inflammatory responses, cell cycle progression, apoptosis, and cell motility (112). Its activation has been shown to upregulate the production of proinflammatory cytokines and has been implicated in various inflammatory diseases (113, 114). Given the established link between inflammation and cancer, MK2 is likely to play a significant role in cancer development and progression (113, 114). Indeed, MK2 inhibition has shown promise in combination with RT to suppress tumor cell growth and reduce metastatic potential. For instance, shRNA-mediated silencing of MK2 in bladder cancer cells has been shown to significantly enhance apoptotic cell death following RT via the upregulation of caspase 3 activity and cytochrome c release (116). Berggren et al. (2019) demonstrated that pharmacologic inhibition of MK2 using PF-3644022 and genetic silencing significantly reduced RT-induced MK2 phosphorylation and proinflammatory cytokine expression (53). Moreover, the combination of PF-3644022 with RT markedly reduced tumor growth and improved survival in patient-derived xenograft models of HNSCC (53). Collectively, these findings suggest that MK2 pathway activation may potentially contribute to RT resistance and that its inhibition could enhance tumor radiosensitivity.

In summary, while both p38 MAPK and its downstream effector MK2 have emerged as promising targets in cancer therapy, their clinical potential remains underexplored. Given their significant roles in regulating inflammation, metastasis, and treatment resistance, further investigation is warranted to validate the therapeutic benefit of targeting these kinases, particularly in combination with RT.

### 3.4 Targeting NF-κB signaling to curb inflammation in cancer

Given the crucial roles of NF-κB in cancer development and progression, targeting it has been extensively explored over the past few decades (27). A few compounds, such as dehydroxy-methyl-epoxy-quinomicin (DHMEQ), fimepinostat (CUDC-907), and dimethylaminoparthenolide (DMAPT), have been proposed as potential radiosensitizers (76–78, 80). These NF-κB inhibitors primarily exert their cytotoxic effects through inducing G2/M cell cycle arrest and inhibiting DNA repair pathways, thereby sensitizing tumor cells to radiation and reducing cell proliferation (76–78, 80). For instance, the combination of DHMEQ, a compound derived from a natural product, with 4 Gy of irradiation produced a synergistic antitumor effect in LNCaP and PC-3 prostate cancer cells by inhibiting radiation-induced NF-κB and upregulating the expression of cell cycle-related proteins, such as p53 and p21 in LNCaP, and 14-3-3σ in PC-3 cells (98). This collectively leads to enhanced G2/M cell cycle arrest (76). *In vivo*, the combination of DHMEQ with IR significantly reduced tumor volume in PC-3 xenograft models compared to either treatment alone (76). Similarly, combining CUDC-907, a dual PI3K/HDAC inhibitor, or DMAPT, an orally active NF-κB inhibitor, with irradiation sensitized glioma and NSCLC cells, respectively, by inhibiting DNA repair (77, 78). In glioma cells, this combination treatment markedly increased p21 expression and reduced phosphorylated CDK1 levels, further enhancing radiosensitivity (77). Collectively, targeting NF-κB, alone or in combination with PI3K/HDAC inhibition, effectively enhances radiosensitivity across multiple cancer types by disrupting DNA repair and promoting G2/M cell cycle arrest, supporting further translational efforts to validate these compounds in clinical settings (77).

Although current evidence supporting the use of NF-κB inhibitors to prevent radiation-induced metastasis is largely confined to preclinical models, several compounds have demonstrated clinical promise based on early-phase safety evaluations. For example, fimepinostat has demonstrated antitumor activity by downregulating p-PI3K downstream targets, increasing histone H3 acetylation, reducing cellular myelocytomatosis (Myc) protein levels, upregulating pro-apoptotic proteins, and inducing G1 cell cycle arrest (115, 116). These effects have been shown to associate with an inhibition of NF-κB signaling (115, 116). Fimepinostat has been investigated in phase I (NCT01742988) and II (NCT02674750) clinical trials in patients with relapsed or refractory diffuse large B-cell lymphoma and high-grade B-cell lymphoma, respectively, demonstrating a durable antitumor response with an acceptable safety profile at 60 mg, administered daily for 5 consecutive days followed by a 2-day break (117, 118). Notably, patients with Myc-altered disease demonstrated an encouraging objective response rate of 64% (7 out of 11 patients), compared to only 29% (2 out of 7 patients) in those with unaltered Myc (119). It is anticipated that the therapeutic efficacy of fimepinostat may be enhanced through combination with other conventional therapies or by selecting patients with Myc overexpression, warranting further investigation.

DMAPT, a water-soluble analog of the natural sesquiterpene lactone parthenolide, has been shown to inhibit NF-κB signaling and eradicate tumor cells in various cancers, including acute myelogenous leukemia, NSCLC, prostate cancer, and PDAC (120–123). Preclinical studies have demonstrated that DMAPT-mediated NF-κB inhibition induces apoptotic cell death by impairing DSB repair, increasing c-Jun phosphorylation, promoting oxidative stress responses, and activating the tumor suppressor p53 (120, 121, 123). Importantly, DMAPT has also been shown to selectively target the tumor cells while protecting neighboring healthy tissues from cancer therapy-induced damage. Morel and co-workers (2017) reported that treatment with DMAPT in a prostate cancer mouse model, administered prior to a combination of low (10 mGy) and high doses (6 Gy) whole-body X-ray, doubled radiation-induced apoptosis in prostate tumor tissue while reducing apoptosis in normal prostate tissues and distant tissues, such as the spleen tissues and colorectal tissues, by 71.7%, 48.2%, and 38.0%, respectively (79). In addition, DMAPT markedly reduces RT-induced fibrosis in normal tissues following long-term exposure to RT, as demonstrated in a preclinical, fractionated irradiation mouse model (124). These findings suggest that combining DMAPT with RT not only enhances the tumor radiosensitivity but may also serve as a radioprotective strategy to minimize RT-induced side effects in normal tissues. Despite its promise, clinical data on DMAPT remain very limited and unpublished to date. A phase I clinical trial conducted by Cardiff University in patients with hematological malignancies demonstrated promising pharmacokinetics with no reported normal tissue toxicity induced by DMAPT (122). Further clinical studies are warranted to validate the therapeutic potential of DMAPT as a radiosensitizer and/or radioprotective agents in well-designed trials, thereby advancing its clinical application.

Another NF-κB inhibitor, DHMEQ, has demonstrated strong anticancer and anti-inflammatory potential in animal models without signs of toxicity (125). Treatment with DHMEQ in both *in vitro* and *in vivo* models of OSCC have been shown to suppress tumor growth and survival in a dose-dependent manner by inducing DNA fragmentation and downregulating anti-apoptotic proteins such as survivin (126). Similarly, DHMEQ exhibited dose-dependent anti-proliferative and anti-invasive effects against NPC cells by inducing G2/M cell cycle arrest and inhibiting TNF-α-induced NF-κB activity (127). Furthermore, the combination of DHMEQ with RT effectively suppressed prostate cancer cell growth and induced G2/M cell cycle arrest compared to RT alone (76). These findings suggest that DHMEQ could potentially overcome radioresistance and RT-induced metastasis, warranting further investigation in early-phase clinical trials to establish a safe and efficacious dose in patients.

In addition to conventional NF-κB inhibitors, several promising repurposed drugs targeting the NF-κB pathway have been identified, including nafamostat mesylate, a synthetic serine protease inhibitor; disulfiram-copper complex, used to treat alcohol use disorder; niclosamide, an anti-helminthic agent; and celecoxib, a non-steroidal anti-inflammatory drug. These agents warrant further investigation for their potential clinical benefits in NF-κB-mediated cancer development (96, 97). For instance, nafamostat mesylate has been shown to inhibit radiation-induced NF-κB activity and exert antitumor effects in gallbladder cancer by promoting apoptosis and enhancing cell cycle arrest (80). *In vivo*, the combination treatment of nafamostat and X-ray (5 Gy) significantly reduced tumor burden without causing body weight loss, indicating a favorable toxicity profile for potential clinical translation (80). Overall, NF-κB inhibitors show promising radiosensitizing potential, particularly by inducing cell cycle arrest and enhancing DNA damage.

## 4 The future with radiogenomics

Despite promising results from preclinical and early-phase clinical studies targeting these pathways in cancers, only a limited number of agents have been successfully translated into clinical applications due to their unclear therapeutic effects and undesirable side effects (70, 71, 83, 111). Furthermore, our understanding of the precise mechanisms underlying radiation-induced inflammation in metastasis and radioresistance remains limited. Since these events are typically mediated by multiple regulatory pathways that vary across cancer types, tailored molecular interventions or combination therapies are likely required for different tumor subtypes. Additionally, tumor heterogeneity further complicates the development of effective and personalized cancer treatments, posing a major challenge to advancing therapeutic strategies (15). Despite the well-established role of online databases on cancer studies, overcoming these challenges will require the integration of advanced technologies coupled with comprehensive databases to decode the complexity of radioresistance and enable the development of safer and more effective therapeutic strategies.

Recent advances in genomic sequencing technologies and image acquisition have already begun to support this effort. These tools enable the identification of prognostic and predictive biomarkers, enhancing early detection, disease prognosis, and treatment selection. In particular, radiogenomics, an emerging tool in precision medicine that leverages artificial intelligence (AI) to correlate genomic alterations (e.g., gene mutations and expression patterns) and imaging features, has gained significant attention in precision oncology (128). By integrating molecular and radiological data, radiogenomics offers deeper insights into tumor biology and tumor heterogeneity, potentially leading to the discovery of optimal biomarkers to improve cancer prognosis and treatment outcomes (128).

In general, the workflow of radiogenomics involves image acquisition, region of interest (ROI) identification and segmentation, quantitative image feature extraction, data mining and informatics analysis, and modeling (128). It relies on high-throughput computing as well as machine learning and deep learning algorithms to manage and analyze a large amount of imaging datasets (129). Compared to traditional imaging approaches, radiogenomics offers a non-invasive and less time-consuming alternative to assist clinicians in early diagnosis and disease monitoring, potentially reducing the risk of recurrence and cancer metastasis. In clinical practice, follow-up imaging after RT is essential for assessing treatment response. Hence, integrating radiomic or radiogenomic analysis into routine imaging workflows can aid in the detection of residual cancer cells and offer insights into resistance mechanisms and therapeutic adaptation (128).

Moreover, the development of AI-powered models enables more accurate characterization of tumor heterogeneity, assesses metastatic potential, and helps uncover clinically relevant imaging signatures that may be imperceptible to the human eye (129). By analyzing radiomic signatures and differentially expressed genes, researchers can gain insight into molecular mechanisms underlying cancer development and progression. For example, Zeng et al. (2021) developed a radiogenomic predictive model to study molecular characteristics and forecast OS of patients with ccRCC by integrating gene expression data from The Cancer Genome Atlas (TCGA) with matched contrast-enhanced computed tomography images from The Cancer Imaging Archive (TCIA) (130). The model demonstrated strong predictive capability in identifying several genetic mutations and mRNA-based molecular subtypes in ccRCC patients, achieving an area under the curve (AUC) ranging from 0.949 to 0.973 (130). By incorporating multi-omics data, such as genomic, transcriptomic, and proteomic information into the radiomic analysis, the model resulted in accurate predictions of OS in ccRCC patients with an AUC of 0.846 compared to 0.755 for the radiomics model alone (130). These findings support the potential of radiogenomics as a non-invasive tool for personalized prognosis and treatment planning, reducing the reliance on invasive tissue biopsies in clinical practice.

**Figure 5:**
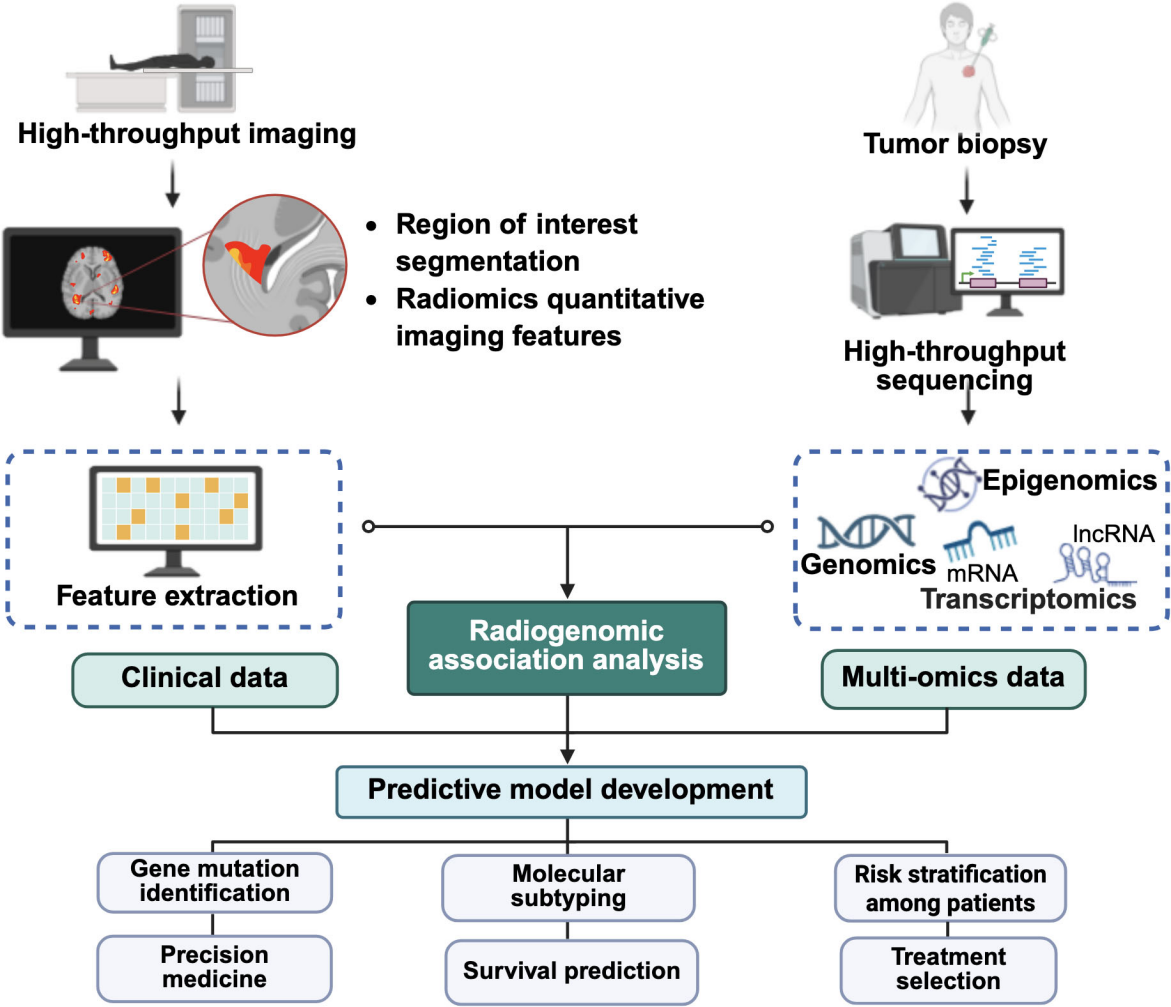
Integration of multi-omics analysis and artificial intelligence in cancer management. The schematic illustrates how medical imaging, clinical, genomic, transcriptomic, and other omics data are integrated and fed into artificial intelligence-driven radiogenomic analysis to develop predictive models with potential applications in gene mutation prediction, survival prognosis, molecular subtyping, treatment risk stratification, and treatment selection. The figure was created using BioRender (https://BioRender.com).

Furthermore, recent advancements in computational methods have positioned the Connectivity Map (CMap), Reactome or Search Tool for the Retrieval of Interacting Genes/Proteins (STRING) (131) as some powerful *in silico* platforms that match disease-specific gene expression signatures with drug-induced transcriptional profiles (132), enabling the identification of compounds capable of reversing pathological expression patterns (131, 132). The integration of radiogenomic approaches with *in silico* platforms is anticipated to offer a cost-effective and scalable strategy for discovering candidate therapeutics targeting radiation-induced metastatic pathways (129, 131). This integration may further advance personalized medicine by aligning targeted therapies with patient-specific molecular profiles, ultimately maximizing therapeutic efficacy (133). **Figure 5** illustrates the integration of radiomic and other omics data (134, 135), which supports the development of predictive models to assess treatment response and metastatic risk following RT.

## 5 Conclusion

RT can inadvertently promote tumor cell survival and metastatic potential and thus a deeper understanding of these mechanisms is important to develop more effective treatments. With the increasing demand for effective cancer treatments, drug repurposing along with the advances in gene sequencing technologies now enable comprehensive tumor profiling, to repositioning drug candidate in cancer treatment. Radiogenomics further offers a non-invasive, cost-effective approach to assess tumor heterogeneity, holding great potential to revolutionize diagnosis, treatment planning, and prognosis. Nevertheless, further research is needed to refine AI algorithms, enhance multi-omics data integration, discover reliable imaging biomarkers, and validate the clinical applicability of radiogenomics across diverse cancer types. Ultimately, these advancements will pave the way for more personalized, efficient, and cost-effective cancer treatment strategies.
